# Predictors of Vaccine Uptake among Migrants in the United States: A Rapid Systematic Review

**DOI:** 10.3390/epidemiologia3040035

**Published:** 2022-10-20

**Authors:** Taysir Al Janabi, Gianna Petrillo, Sunny Chung, Maria Pino

**Affiliations:** New York Institute of Technology, College of Osteopathic Medicine (NYITCOM), Glen Head, NY 11545, USA

**Keywords:** COVID-19, migrant, refugee, vaccine, vaccine uptake, predictor, determinant, US

## Abstract

Evaluating challenges to vaccine uptake in non-US-born individuals is necessary for increasing national vaccination rates. This rapid review was conducted to investigate predictors of vaccine utilization among US migrants. The Preferred Reporting Items for Systematic Review and Meta-Analysis (PRISMA) checklist was utilized, along with the Rayyan webtool, to facilitate the process of identifying primary research articles. Data were independently extracted by using a piloted, customized form. This was tabulated and the results were reported. Of the 186 abstracts reviewed, nine articles were included. Populations included in this review were refugees (*n* = 1), undocumented migrants (*n* = 1), migrants crossing the US–Mexico border (*n* = 2), Blacks (*n* = 1), and US-born vs. non-US-born adults (*n* = 1). Three studies focused on “foreign-born” children. The vaccines included in the literature reviewed were both combined series and individual, with one study addressing immunization instead of specific vaccines. Detailed characteristics of these studies and their quality evaluations were also reported. This review identified gaps in research regarding immunization among different migrant groups. Multilevel interventions should be considered to leverage the existing facilitators and address the known modifiable barriers to creating an accessible and supportive environment for marginalized populations.

## 1. Introduction

Vaccinations remain a simple, safe, and effective method for the protection of both individual and global health. Yet, the success of a vaccine’s uptake will continue to depend on its availability, coverage, and acceptance by the greater population. In 2019, the World Health Organization (WHO) named vaccine hesitancy as one of the top ten global health threats [1]. In past years, the United States (US) has experienced its own reduction or delay in vaccinations, leading to outbreaks of once preventable, communicable diseases, such as measles and mumps, especially in the pediatric population [2]. More recently, this pattern of vaccine uncertainty was observed during the distribution of the COVID-19 vaccines against SARS-CoV-2. Individual concerns for vaccine-associated risks, unknown vaccine efficacy and scientific technology, loss of autonomy, political affiliation, combined with mistrust for the medical profession were among the main reasons provided for COVID-19 vaccine refusal [3]. In addition, there is a history of racial discrimination and negative experiences within the US health care system which has greatly contributed to vaccine hesitancy among ethnic minorities [4]. While these factors have been cited in these and other works that study vaccine uptake among US citizens [3], vaccination uptake patterns continue to be investigated in the migrant population and their children [5].

The Centers for Disease Control and Prevention (CDC) continues to promote and improve the health of immigrants, refugees, and migrants, while preventing the importation of infectious diseases and other conditions of public health significance into the US by these groups. This includes mandatory health screenings for all immigrants and refugees entering the US [6]. A medical examination is mandatory for all applicants outside the US applying for an immigrant visa and for all refugees coming to the US. Outside the US, medical examinations are performed by approximately six hundred physicians known as panel physicians [7]. In 2013, the CDC partnered with the Department of State to implement the Vaccination Program for US-bound Refugees [8]. This program intended to improve the health of individuals intending to establish residence in the US while reducing the outbreaks of vaccine-preventable diseases by offering the first dose of standard, age-appropriate vaccinations, such as polio; measles, mumps, and rubella (MMR); Hemophilus influenza type b (Hib); and tetanus [8].

While these mandates have been beneficial to public health, challenges to vaccination continuity do exist for all. In a study performed by Kimmel et al., barriers to immunization included systems barriers (inadequate organization of the health care system), health care provider barriers (clinicians not adequately educated about vaccines), and parent and patient barriers (fear of immunization-related adverse events) [9]. However, the most significant systems barrier to immunization is supply and distribution [10]. Other limitations to vaccine uptake cited in migrant populations include the real or perceived cost of receiving vaccinations; language, religious, and cultural barriers; and limited opportunities to ask relevant questions to health care professionals [5].

Access to all required vaccinations among all non-US-born populations and their families as they acclimate to living in a new country is critical for both these individuals and the public. To achieve this, barriers to vaccine uptake in these populations require a thorough assessment so that new recommendations and ways to enhance vaccine availability can be decided upon. This rapid review was conducted to investigate the predictors of vaccine uptake among US migrants, as the nation continues to navigate through the COVID-19 pandemic, while managing the most recent outbreaks of monkeypox and polio in its larger cities [11].

## 2. Materials and Methods

Due to the nature of this study, approval by the Institutional Review Board was not required. This study used the Preferred Reporting Items for Systematic Review and Meta-Analysis (PRISMA) checklist to guide this rapid review [12]. The research team registered the review with the International Prospective Register of Systematic Reviews (PROSPERO: CRD42022333025, York, UK).

### 2.1. Inclusion, Exclusion, and Data Extraction

**Figure 1:**
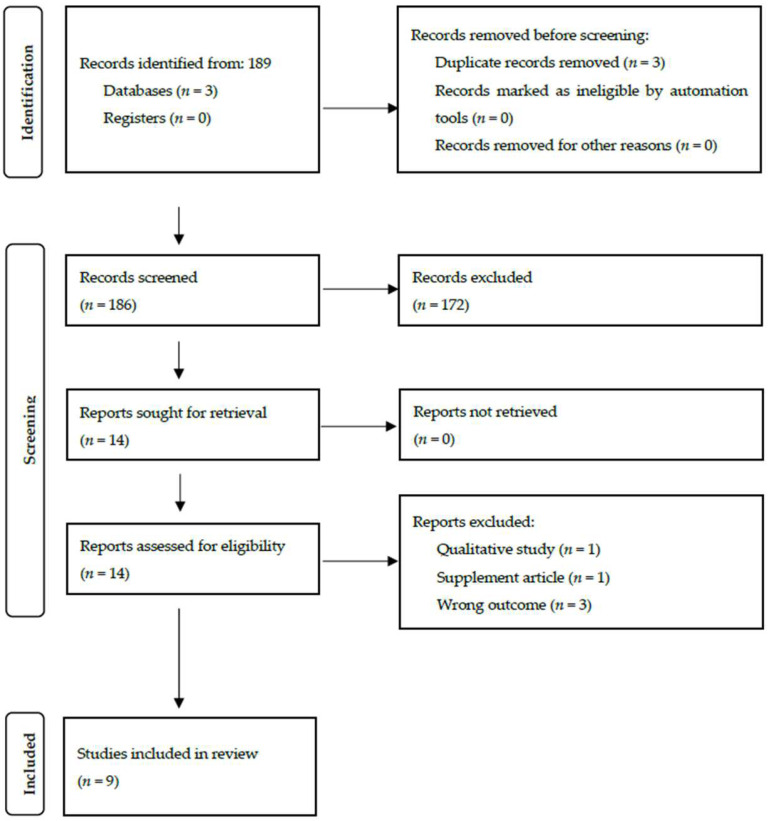
Study selection process shown by PRISMA 2020 flow diagram for new systematic reviews including searches of databases, registers, and other sources.

We limited our search to include primary research and excluded (1) commentaries, (2) editorials, (3) non-peer-reviewed articles, and (4) case reports. Titles and abstracts of all studies were independently reviewed by three investigators using the Rayyan web tool and discrepancies were resolved through reviewer discussion and consensus. Full texts for the studies that met initial inclusion criteria were obtained and reviewed. The inclusion and exclusion criteria were created using a PICOS framework [13]. Table 1 summarizes the criteria. The review process in this rapid review is detailed by the PRISMA flow diagram and outlined in Figure 1.

### 2.2. Search Strategy

The search was performed in May 2022 and included studies published from 1 January 1990 to 1 May 2022. The literature search was conducted in PubMed, Cumulative Index to Nursing and Allied Health Literature (CINAHL), and Cochrane. The Cochrane database was searched for grey literature. A search was performed using the focused key medical subject heading (MeSH) terms for: “vaccine uptake“, “refugee,” “United States,” and “facilitators/barriers.,” see Supplementary Materials S1. The search strategy adapted in PubMed was applied to the other two databases. Reference List Searching (Snowballing) was also performed. Results were filtered by the English language based on database features. Cochrane does not have an English language filter. Results were imported into Endnote and deduplicated by an independent reviewer. Once completed, the remaining articles were imported into Rayyan, a free web-based systematic review tool where the team performed an initial eligibility screening based on title and abstracts. Titles and abstracts were screened based on inclusion and exclusion criteria. In total, 186 abstracts were evaluated by the reviewers. Initially, 176 records were excluded after reviewing abstracts. Ten full-text articles were obtained, and eight articles were excluded after a full-text review. Because of the limited number of articles left for analysis, the research team decided to expand the review to include migrants in the inclusion and exclusion criteria instead of only refugees. The change was updated with PROSPERO; however, the search strategy remained the same since the text words “migrant” and “migrants” were included in the search strategy of all databases.

### 2.3. Data Extraction

Data were independently extracted by two reviewers using a customized form, which was piloted for this review, including study author(s), year(s) of study, study design, method, vaccine type, study population, total sample size, total migrant sample size, objective addressed, and predictors identified. Discrepancies at any stage were resolved by consensus. Table A1 outlines the characteristics of the included studies in this review.

### 2.4. Risk Assessment

The quality of all included studies was independently assessed by two reviewers using a tool with ad hoc criteria to identify all important potential sources of bias; criteria included are random sampling method, sample size, face/construct validity of the survey, and the appropriateness of the statistical methods, see Table A2. Assessed studies were given an overall rating of low, medium, or high concerns. Data were not excluded based on study quality; however, the data were used to supplement the narrative synthesis and discussion. The overall quality of this review was assessed using the Risk of Bias in Systematic Reviews (ROBIS) tool [14], see Supplementary Materials S1.

### 2.5. Data Synthesis and Analysis

Extracted data were tabulated and results presented as reported in the studies. All data were synthesized narratively. Quantitative data addressing the determinants of vaccine uptake were tabulated by the population and vaccine type. Data synthesis and analysis were carried out by two reviewers.

## 3. Results

**Figure A1:**
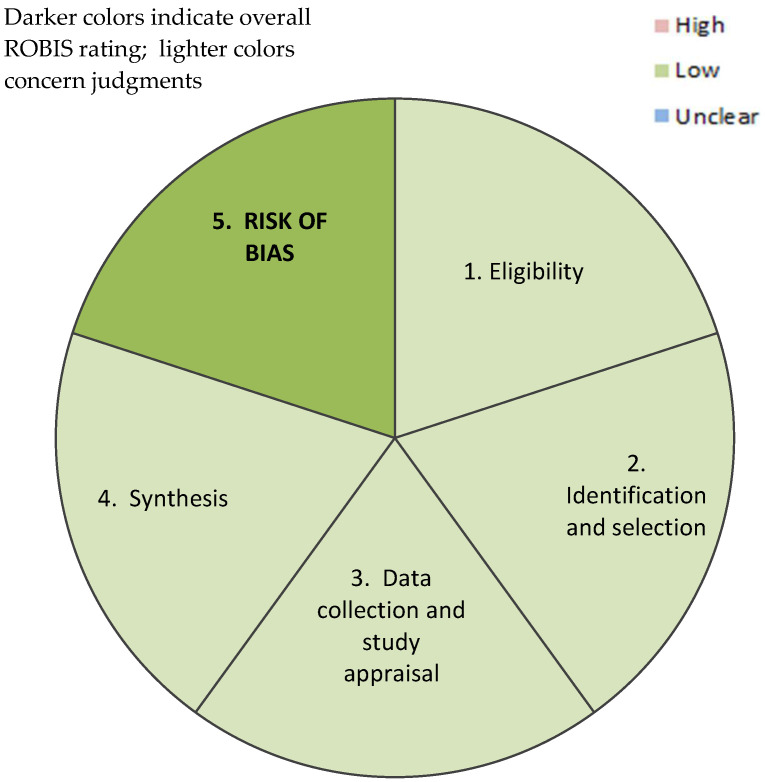
Graphical presentation of ROBIS evaluation results from this review.

Out of the 186 abstracts that were reviewed after updating the protocol, 172 records were excluded. Fourteen full-text articles were obtained, and five articles were excluded after a full-text review. Nine articles were included in this review [15]. Three studies focused on “foreign-born” children or children of non-US-born mothers, and five papers focused on adults. Lastly, one study was for individuals 16 years of age and older. Populations included in the review were refugees (*n* = 1), undocumented migrants (*n* = 1), migrants crossing the US–Mexico border (*n* = 2), Blacks (*n* = 1), and US-born vs. non-US-born adults (*n* = 1). Three studies reported on seasonal flu vaccine; two studies investigated COVID-19 vaccines; one study described the combined 4:3:1:3:3 series—four doses of diphtheria, pertussis, and tetanus; three doses of inactivated polio vaccine (IPV); one dose of MMR; three doses of Hib vaccine; and three doses of hepatitis B vaccine (HepB). One study reported on the combined 4:3:1:3*:3:1:4 series (≥4 doses of diphtheria, tetanus, and acellular pertussis vaccine (DTaP)/diphtheria and tetanus toxoids vaccine/diphtheria, tetanus toxoids, and pertussis vaccine, ≥3 doses of IPV, ≥1 dose of measles-containing vaccine, ≥3 or ≥4 doses of Hib vaccine (depending on product type of vaccine; denoted as 3* in the series name), ≥3 doses of HepB vaccine, ≥1 dose of varicella vaccine (VAR), and ≥4 doses of pneumococcal conjugate vaccine, PCV); one study examined the human papilloma vaccine (HPV) initiation. Another paper reported on individual vaccines: DTaP, IPV, HepA, HepB, Hib, meningococcal vaccine (MCV), MMR, PCV, rotavirus (RV), and VAR. One article reported on the H1N1 pandemic influenza vaccine; one more study addressed immunization rather specific vaccines. Eight studies were cross-sectional in design, and one was a longitudinal study. Detailed characteristics of included studies are shown in Appendix A and their quality evaluation is displayed in Appendix B. The ROBIS evaluation results reveal that this review contained a low risk of bias in the following categories: eligibility, identification and selection, data collection and study appraisal, synthesis, and overall (see Figure A1).

Sun et al., Varan et al., and Buelow and Van Hook investigated determinants of vaccination among children [15]. Sun et al. assessed childhood immunization in general (no specific vaccine). Varan et al. evaluated DTaP, IPV, HepA, HepB, Hib, MCV, MMR, PCV, RV, VAR, and the combined 4:3:1:3*:3:1:4 series. The study reported that foreign-born children had significantly lower vaccination coverage for DTaP, HepA, HepB, Hib, PCV, and RV. The study also observed that the completion rates for the 4:3:1:3 and the 4:3:1:3*:3:1:4 series were 48% and 26.7%, respectively, which were lower than the rates of the US-born children [16]. Buelow and Van Hook assessed the timely completion of the combined 4:3:1:3:3 series, which was only 41% for children of native-born parents [17].

All three studies identified the following predictors, which were significantly associated with lower vaccine uptake among migrant children: the lack of health insurance, non-English language speaker, lower family income, non-first-born child, more than one health care provider, recent migration to the US, foreign-born mother, and non-US citizen mother.

The same studies also identified the following predictors, which were significantly associated with high vaccine coverage among migrant children: US citizenship, longer residential duration in the US, more than 12 years of maternal education, child older than 24 months, higher income, the residence of Midwest and southern region, and the availability of health insurance. Additionally, non-Hispanic Asian children are more likely to have their vaccination series completed compared to other migrant children. Moreover, the Spanish language during the study interview was significantly associated with high vaccine uptake.

Two cross-sectional studies explored the COVID-19 vaccine uptake in two populations; Zhang et al.’s study among refugees and Page et al. among undocumented populations [18]. Among refugees, those who identified as male and those who worked in essential jobs are more likely to get vaccinated. Among undocumented migrants, female gender, increased age, comorbidities, and positive attitudes towards vaccination in general, and the COVID-19 vaccine, specifically, are significantly associated with increased COVID-19 vaccine uptake.

Rodriguez-Lainz et al. and Ejebe et al. reported that the seasonal flu vaccine coverage was 33.7% in 2012 and 18.6% in 2013, respectively [19]. Chuey et al. reported that the seasonal flu vaccine coverage for foreign-born adults was lower than their US-born counterparts for most of the influenza seasons examined [20]. All the above-mentioned studies also observed that seasonal flu vaccine uptake was significantly associated with nativity, US citizenship, comorbidity, health insurance, and age [19,20,21]. The observed increase in the vaccine uptake was associated with those who were born in the US—those with Asian ethnic background being the most likely to get their flu shot among other US-born minorities—foreign-born Hispanic, older than 60 years old, those with health insurance, and those with comorbidities. For migrants who crossed the US–Mexico border, the flu vaccine uptake was significantly associated with the frequency of border crossing with a cut-off of eight times per month. Those who crossed the border more than eight times per month were less likely to receive their flu shot. Rodriguez-Lainz et al. also reported that the lower H1N1 vaccine uptake in the adult Mexican migrants crossing the border in California and Texas in 2010 was significantly associated with residence on the US side of the border, those with higher than high school education, and those who commute more than eight times per month [19].

The Cofie et al.’s cross-sectional study explored predictors of HPV vaccine initiation among Black adults (age = 18–37 years). It was observed that HPV vaccine initiation was only 21.7% [5]. The study also reported that non-US-born Black populations are less likely to initiate HPV vaccination. However, after adjusting for health-related factors, this demographic became insignificant. Additionally, the place of birth significantly predicted HPV initiation. The strongest predictability was associated with the US, Africa to a lesser extent, and the least is the Americas/Caribbean Islands. Moreover, having some college degree and the younger age at which the vaccine was administered are significant predictors of HPV initiation. Females were a significant predictor in all racial groups.

## 4. Discussion

Migrants are considered underserved populations, especially in the health care system. These individuals and their families often live in poor situations and have limited access to health services [22]. Additionally, they often lack data that guide interventions and health policies [23,24]. The review highlights the underimmunization in the migrant populations in the US, which was consistent with the literature. Many studies have documented that reduced vaccination uptake often occurs among individuals with migrant backgrounds and those who speak a language other than English at home [25].

### 4.1. Childhood Immunization

The predictors of low childhood immunization identified by Sun et al., Varan et al., and Buelow and Van Hook might suggest the lack of accessibility or inconsistency in accessing health care for migrant children resulting in incomplete immunization. The significant association of low income with decreased vaccine uptake might be explained by the misconception that vaccines are not free, while the association between being a second child and underimmunization might be explained by parental distraction with having more than one child [26].

Non-Hispanic Asian children are more likely to have their vaccination series completed compared to other migrant children, a finding reported by other studies in New Zealand and Canada, where children of Asian ethnicity were more likely to be up to date with their immunization [25]. The higher educational levels of mothers have been identified as a positive predictor of vaccine completeness among migrant children [27]. Highly educated mothers might communicate effectively with their child’s health care providers and understand the importance of immunization and vaccine completeness.

### 4.2. COVID-19 Vaccine

High COVID-19 vaccine uptake among refugees was predicted by being male and being an essential worker. These findings could be due to individuals in these positions being in constant contact with customers, who might have different beliefs and levels of perceived risk. Subsequently, this increases essential workers’ risk of contracting COVID-19 by not adhering to the preventive measures. Studies have observed that increased perceived COVID-19 risk is associated with positive attitudes toward COVID-19 vaccination among refugees [28]. Moreover, refugees might not have many opportunities to work remotely as in other professions. Gender is a controversial determinant among refugees. One study found that gender did not predict vaccine uptake among refugees in Australia [29], while another observed that the female gender was a predictor of COVID-19 vaccine uptake among refugees in Lebanon [30]. More research will be necessary to clarify the role of gender in COVID-19 vaccine uptake among this population.

High COVID-19 vaccine uptake among undocumented migrants was predicted by the female gender, increased age, comorbidities, and positive attitudes toward vaccination. The female gender has been reported to be a significant predictor of COVID-19 vaccine uptake among undocumented migrants in the US [31]. Other studies reported similar findings of increased perceived COVID-19 risk with comorbidities and older age among the nonmigrant populations [32]. Undocumented migrants account for up to 40% of human mobility worldwide through unofficial channels, which leaves them without stable access to health care. Additionally, undocumented migrants often mistrust the governments of the host countries, preventing them from accessing the health system as they fear being reported to the immigration authorities [33].

### 4.3. Seasonal Flu Vaccine

The predictors of seasonal flu vaccine uptake reported by Rodriguez-Lainz et al. and Ejebe et al. might be explained by the fact that the flu virus has more severe consequences in the elderly population and those with comorbidities than in others [34]. Moreover, the time constraints associated with frequent commutes across the border might leave preventive care as a secondary issue. Moreover, migrant workers are less likely to have typical paid medical or family leave [35]. It was reported also by Ejebe et al. that only 31.2% of migrants with a chronic health condition and only 28.4% of those over age 60 were estimated to have received their seasonal influenza vaccination. Tailored educational campaigns toward this population and allocating more resources might be necessary to improve the vaccination rate, such as the utilization of community health workers (CHWs) in developing and implementing educational workshops. Additionally, health policies should encourage flu vaccination among migrants who cross the border more than eight times per month to increase the vaccination rate, perhaps with incentives. The findings of Chuey et al. were consistent with the literature as being a US-born Asian and foreign-born Hispanic significantly predicted the seasonal flu vaccine uptake in the US [36]. Hispanics might have well-established social ties in the US facilitating vaccination compared to other foreign-born migrant populations. Further research is required to explore the influence of culture and social norms on vaccination among minorities.

### 4.4. H1N1 Influenza Virus Vaccine

The predictors of H1N1 vaccine uptake identified by Rodriguez-Lainz et al. might be explained by the fact that frequent commutes might limit individuals from seeking vaccination. Additionally, those on the US border may not be familiar with the locally available resources to get vaccinated. Moreover, language might be a barrier.

### 4.5. HPV Vaccine

The predictors of HPV vaccine initiation among Black adults reported by the Cofie et al.’s cross-sectional study may point to some accessibility barriers among this group, such as language, documentation, and transportation. A misconception about HPV vaccine safety, sociocultural values, and health care providers’ recommendations could influence the HPV vaccine uptake. Research has reported that primary care physicians serving minorities do not recommend HPV vaccination routinely [37]. Active engagement of school and primary care physicians, with HPV vaccine promotion campaigns, may improve vaccine uptake. Additionally, the younger age at which the vaccine is administered is a significant predictor of HPV initiation. Similar findings were reported by Pérez et al. [38], highlighting the importance of communication to this population at a younger age to enhance HPV vaccine uptake coverage.

#### Limitations

This rapid review has limitations. First, the limited number of included databases might not have provided a comprehensive search, as the authors could have missed some relevant studies. Secondly, the quality of the included studies varied. For example, the study performed by Sun et al. had the *p*-value set to 0.10, and the statistical tests were run multiple times, which might create a type I error. Another example is the Zhang et al., study which had some concerns about the response rate. This could pose a question regarding the external validity of the study. In addition, the lack of interrater reliability of participant responses for assignment to categories could be a concern for internal validity of the study. Additionally, the findings might not be generalizable because of the snowballing sampling method. Thus, potential bias might have been introduced in the study.

The findings of Rodriguez-Lainz et al. should be interpreted carefully as it is unknown if the survey was validated by a pilot study or other means of verification. Additionally, collapsing data from multiple sites without weighing the data or correcting for multiple comparisons might undermine the significance of the results. The results of the Chuey et al. could be weakened by the number and types of the statistical tests run, including linear regression and *t*-tests, without verifying the assumptions of those tests. The data was also subdivided many times, which could make the few significant results questionable given the number of categories and comparisons.

Thirdly, the diversity of migrant groups, the different types of vaccines, variable sample sizes, and different time periods could have limited the comparability of the findings. Lastly, some studies did not make separate analyses between migrant and nonmigrant populations. However, they included samples of US- and non-US-born individuals, resulting in difficulty in identifying migrant-specific group findings. Future studies should take caution with some of these limitations mentioned in the included studies.

## 5. Conclusions

This review identifies gaps in research regarding immunization among different migrant groups, which are part of the World Health Organization (WHO) vision of the Immunization Agenda (IA2030) [39]. Multilevel interventions should be considered to leverage the existing facilitators and address the known modifiable barriers to creating an accessible and supportive environment for marginalized populations, including consistent health policies to mobilize resources toward the health of migrant populations [40]. For example, policies that endorse educational and employment opportunities for mothers might improve childhood vaccination by offering stable health insurance coverage through employment. Other strategies to mitigate the impact of underimmunization among these populations may include mobilizing CHWs or volunteers to establish mobile vaccination clinics in the areas where migrant populations live, work, and study to provide vaccination and educational materials [41]. Research has shown that mobile clinics could be cost-effective and improve health outcomes of underserved populations [42]. Mobile clinics could enhance COVID-19 vaccination among migrant populations, as this option might help overcome the public transportation limitation, reduced digital literacy, and widespread misinformation about vaccination. Another approach would be reaching out to families through Special Supplemental Nutrition Program for Women, Infants, and Children (WIC), a nutritional support program for low-income families regardless of their immigration status, which also offers immunization screening and referrals [43]. WIC has helped to improve vaccination coverage among low-income families [44]. Additionally, targeted culturally and linguistically appropriate messages could be considered to increase knowledge and awareness of specific vaccines and populations [45]. Moreover, disseminating vaccine-related information to health care professionals is essential to increase vaccine confidence [46], especially in the context of the COVID-19 and HPV vaccines.

## Figures and Tables

**Table 1:** Inclusion and exclusion criteria using PICOS framework.

PICO	Inclusion Criteria	Exclusion Criteria
Population	Adult, adolescent, and child migrants (foreign-born) and children of migrants (under 18 years of age, with at least one migrant parent) residing in the US.	Migrant is not defined by country of origin or birth; data were not collected in the US; data were not collected within the specified timeframe; articles that are not primary research; articles not written in English.
Intervention	Predictors that significantly assess the COVID-19 vaccine and other vaccine uptake in migrant populations will be examined. Papers about variables that assess the likelihood of accepting COVID-19 vaccines and other vaccines among migrant populations will be considered (e.g., country of origin, age, income, integration into society, health belief, etc.).	This review will not consider interventions that influence vaccination.
Comparison	No control was selected for this review.	Not applicable.
Outcomes	Determinants of vaccine uptake in migrant populations.	Not applicable.

## Data Availability

Not applicable.

## Supplementary Materials

The following supporting information can be downloaded at: https://www.mdpi.com/article/10.3390/epidemiologia3040035/s1, Supplementary File: S1.

## Appendix A

**Table A1:** Detailed characteristics of the included studies in the review.

Study Author(s)	Year(s) of Study	Study Design	Method	Vaccine	Study Population	Total Sample Size	Total Migrant Sample Size		Objective Addressed	Predictors Identified
						(*n*)	(*n*)	(%)		
Sun et al.	1996	Cross-sectional	Survey	No particular vaccine noted	Children born in the US- vs. non-US-born	270	211	78.1	Vaccine uptake among US vs. non-US-born children in NYC	US residential duration (+) ^1^ Non-English primary language (−) Health insurance (+) Family Income (+) Immigration status (−) ^2^
Page et al.	2021	Cross-sectional	Survey	COVID-19	Undocumented migrants Age ≥ 16 years based in: US, Switzerland, Italy, and France	812	142 from Baltimore 441 from Geneva 126 from Milano 103 from Paris	17.5 54.3 15.5 12.7	Perceived Accessibility Drivers and barriers for Demand	Gender (female) (+) Age (+) Comorbidity (+) Attitudes towards vaccination (+)
Victoria H. Buelow and Jennifer Van Hook	2007	Cross-sectional	Looking at childhood immunization from 2000–2003 (NHIS)	Combined 4:3:1:3:3 series ^3^	Children 19 months to 5 years of the US-born mothers vs. non-US-born mothers who live with mothers and have exact dates of vaccination	3947	1227 children of foreign-born mothers	31.1	The influence of parental immigration status, citizenship, and the residential duration on the completion of the childhood immunization series	Parental nativity (“composition”) (−) US citizenship (+) US residential duration (+) Health insurance (+) Socioeconomics (−)
Ejebe et al.	2013	Cross-sectional	Survey	Seasonal influenza	Adult Mexican migrants crossing at key transit points in Tijuana, Mexico	2313	2313	100	The uptake of the seasonal flu vaccine uptake	Gender (female) (+) comorbidity (+) Health insurance (+)
Zhang et al.	2020–2021	Cross-sectional	Survey	COVID-19	Adult refugees	435	166 from Bhutan 113 from Somalia 68 from Afghanistan 39 from South Sudan 34 from Burma/Myanmar	38.2 26.0 15.6 9.0 7.8	The acceptance of the COVID-19 vaccine and determinants of its uptake	Gender (male) (+) Employment as an essential worker (+)
Varan et al.	2016	Cross-sectional	Analysis of the National Immunization Survey 2010–2012 that assesses vaccination coverage among US children aged 19–35 months	Individual vaccines ^4^ and Combined 4:3:1:3*:3:1:4 series ^6^	Children aged 19–35 months born in the US vs. non-US-born	52,441	491	0.94	Vaccination rate in the US children aged 19–35 months among different demographic characteristics	Nativity (−) Ethnicity/race (+) ^5^ Age of child (+) Study interview language (+) ^7^ Maternal education (+) Income: Poverty ratio (+) Midwest/ Southern region of residence (+) Health insurance (+) Birth order (−) Multiple health care providers (−)
Rodriguez-Lainz et al.	2010 and 2012	Cross-sectional	Survey	H1N1 pandemic influenza vaccine in 2010 Seasonal flu vaccine in 2012	Adult migrant crossing the US–Mexico border in 2010 and 2012 in CA and TX	559 participants in 2010 1423 participants in 2012	559 1423	100 100	The uptake of the H1N1 pandemic flu vaccine in 2010 and seasonal flu vaccine in 2012 among the US–Mexico border crossers	Regarding H1N1 flu vaccine in 2010: Level of education (−) Residence location (Mexico vs. US border) Regarding seasonal flu in 2012: Comorbidity (+) Health insurance (+) Regarding both vaccines: Age (+) Frequency of border crossing (−)
Cofie et al.	2019	Cross-sectional	Analysis of pooled 2013–2017 National Health Interview Survey (NHIS)	HPV vaccine initiation	Black adults aged 18–37 years (the US-born vs. non-US-born; the study did not specify the country of birth)	5246	(*n*) was not specified Africa Mexico/ Caribbean Islands/South America	54.5 33.1	The HPV vaccine initiation among Black adults 18–37 years (the US-born vs. non-US-born) and its associated predictors	Gender (female) (+) Country of birth (+) ^8^ College degree (+) Age at HPV vaccine initiation (−)
Chuey et al.	2021	Longitudinal study	Analysis of pooled 2012–2013 to 2017–2018 National Health Interview Survey (NHIS)	Seasonal flu	Adults (the US-born vs. non-US-born)	2012–2013 31,077 2013–2014 33,126 2014–2015 32,790 2015–2016 29,345 2016–2017 27,279 2017–2018 24,495	5989 6100 6067 4832 3936 3732	17.7 18.1 18.5 18.9 18.7 18.7	The seasonal flu vaccine uptake among adults in the US using NHIS 2012–2018	US citizenship (+) Nativity (−) ^9^

^1^ (+) means positive correlation. ^2^ (−) means negative correlation. ^3^ 4:3:1:3:3 vaccine series include 4 doses of diphtheria, pertussis, and tetanus, 3 doses of polio vaccine, 1 dose of measles, mumps, and rubella, 3 doses of Hemophilus influenza type b vaccine, and 3 doses of hepatitis B vaccine. ^4^ Individual vaccines include DTaP, IPV, HepA, HepB, Hib, MCV, MMR, PCV, RV, VAR. ^5^ Only non-Hispanic Asian and non-Hispanic other. ^6^ 4:3:1:3*:3:1:4 vaccine series include ≥4 doses of diphtheria, tetanus, and acellular pertussis vaccine/diphtheria and tetanus toxoids vaccine/diphtheria, tetanus toxoids, and pertussis vaccine, ≥3 doses of poliovirus vaccine, ≥1 dose of measles-containing vaccine, ≥3 or ≥4 doses of Hemophilus influenzae type b vaccine (depending on product type of vaccine; denoted as 3* in the series name), ≥3 doses of hepatitis B vaccine, ≥1 dose of varicella vaccine, and ≥4 doses of pneumococcal conjugate vaccine. ^7^ If the study interview was conducted in Spanish. ^8^ If the place of birth was the US. ^9^ Foreign-born Hispanics were the only foreign-born minority group significantly associated with increased seasonal flu throughout the study period compared to the non-US-born individuals.

## Appendix B

**Table A2:** Quality assessment of the included studies in the review.

Study	Random Sampling Method	Sample Size	Face/Construct Validity of the Survey	Appropriate Statistical Methods	Overall Rating	Comments
Sun et al.	Low concern	Low concern	Low concern	High concern	Low concern	The *p*-value was set to 0.10, and the tests were run multiple times, which might create type I error.
Page et al.	Medium concern	Medium concern	Low concern	Medium concern	Medium concern	The frequency of running the statistical tests might undermine the significance of some of the assessed variables. Additionally, the nonrandom sampling could lead to potential bias or issues with external validity.
Victoria H. Buelow and Jennifer Van Hook	Low concern	Low concern	Low concern	Low concern	Low concern	Not applicable.
Ejebe et al.	Low concern	Low concern	Low concern	Low concern	Low concern	Not applicable.
Zhang et al.	Medium concern	Low concern	High concern	Medium concern	Medium concern	The snowball sampling method with no response rate and no apparent check of interrater reliability (if there were even multiple raters) of participant responses for assignment to categories leads to concern about dependency of participants and potential bias in results.
Varan et al.	Low concern	Low concern	Low concern	Low concern	Low concern	Not applicable.
Rodriguez-Lainz et al.	Low concern	Low concern	Medium concern	High concern	Medium concern	Little verification of the survey itself along with collapsing data from multiple sites without weighing the data or correcting for multiple comparisons undercuts the significance of the results.
Cofie et al.	Low concern	Low concern	Low concern	Low concern	Low concern	Not applicable.
Chuey et al.	Low concern	Low concern	Low concern	High concern	Medium concern	Many types of tests run including linear regression and t-tests without any verification of the assumptions of those tests. Data was subdivided many times, which makes the few significant results questionable given the number of categories and comparisons.
