# Supplementary material for: Predictors of Vaccine Uptake among Migrants in the United States: A Rapid Systematic Review
Source: Epidemiologia (Basel). 2022 Oct 20;3(4):465–81. doi: 10.3390/epidemiologia3040035 (PMC9777676; doi:10.3390/epidemiologia3040035)
Supplement: Supplementary file 1 [file epidemiologia-03-00035-s001.zip › epidemiologia-1920919-supplementary.pdf]

Research Question	Databases and Resources	Limits
What are the predictors of vaccine uptake among refugees in the United States?	**Select Core Databases:**<br>PubMed<br>CINAHL<br>Cochrane (for grey literature)<br><br>Reference List Searching (Snowballing)	Date: January 1, 1990, May 1, 2022<br>Age Range: all<br>Language: English<br>Inclusion: Primary Research<br>Exclusion: reviews, commentaries, editorials, non-peer-reviewed articles, case reports

Database: PubMed

	**Concept: Vaccine Uptake**	**Concept: Refugee**	**Concept: U.S.**	**Concept: Facilitators/Barriers**
**Thesaurus Terms/ Subheading s**	“Vaccination/psychology” [MeSH] OR<br>“Vaccination” [MeSH] OR<br>“Vaccination/methods” [MeSH] OR<br>“Vaccination/standards” [MeSH] OR<br>Vaccination/statistics & numerical data* [MeSH] OR<br>“Immunization” [MeSH] OR	“Refugees” [MeSH] OR<br>“Refugees*” [MeSH] OR<br>Refugee/statisitcs & numerical data* [MeSH] OR<br>Transients and Migrants/statistics & numerical data* [MeSH] OR	“United States” [MeSH] OR	“Attitude” [MeSH] OR
**Textwords**	“Vaccination” OR<br>“Vaccinations” OR<br>“Vaccine acceptance” OR<br>“Vaccination coverage” OR<br>“Vaccination coverage immunization” OR<br>“Immunization willingness” OR<br>“Immunization, active” OR<br>“Active immunization” OR<br>“Active immunizations” OR<br>“Immunizations, active”	“Refugee” OR<br>“Refugees” OR<br>“Political asylum seeker” OR<br>“Asylum Seeker, Political” OR<br>“Asylum Seekers, Political” OR<br>“Seekers, Political Asylum” OR<br>“Political refugees” OR<br>“Political refugee” OR<br>“Refugee, political” OR<br>“Refugees, political” OR<br>“Asylum Seeker” OR<br>“Asylum Seekers” OR<br>“Seeker, asylum” OR<br>“Seekers, asylum” OR<br>“Displaced persons” OR<br>“Displaced person” OR<br>“Person, displaced” OR	“United States of America” OR<br>“United States OR<br>“America” OR<br>“U.S.” OR<br>“US” OR<br>“USA” OR<br>“U.S.A.”	“Barrier” OR<br>“Barriers” OR<br>“Facilitator” OR<br>“Facilitators” OR<br>“Determinant” OR<br>“Determinants” OR<br>“Factor” OR<br>“Factors” OR<br>“Influence” OR<br>“Influences” OR<br>“Attitude” OR<br>“Attitudes”

		"Persons, displaced" OR<br>"Internally displaced persons" OR<br>"Displaced person, internally" OR<br>"Displaced persons, internally" OR<br>"Internally displaced person" OR<br>"Transients and migrants*" OR<br>"Migrant" OR<br>"Migrants"<br>Migrants		

Line 1: "Vaccination/psychology" [MeSH] OR "Vaccination" [MeSH] OR "Vaccination/methods" [MeSH] OR "Vaccination/standards" [MeSH] OR Vaccination/statistics & numerical data* [MeSH] OR "Immunization" [MeSH] OR Vaccination OR Vaccinations OR Vaccine acceptance OR Vaccination coverage OR Vaccination coverage immunization OR Immunization willingness OR Immunization, active OR Active immunization OR Active immunizations OR Immunizations, active
Results: 522,344

Line 2: "Refugees" [MeSH] OR "Refugees*" [MeSH] OR Refugee/statisitcs & numerical data* [MeSH] OR transients and Migrants / statistics & numerical data* [MeSH] OR Refugee OR Refugees OR Political asylum seeker OR Asylum Seeker, Political OR Asylum Seekers, Political OR Seekers, Political Asylum OR Political refugees OR Political refugee OR Refugee, political OR Refugees, political OR Asylum Seeker OR Asylum Seekers OR Seeker, asylum OR Seekers, asylum OR Displaced persons OR Displaced person OR Person, displaced OR Persons, displaced OR Internally displaced persons OR Displaced person, internally OR Displaced persons, internally OR Internally displaced person OR Transients & migrants* OR Migrant OR Migrants
Results: 27,460

Line 3: Line 1 + Line 2
Results: 808

Line 4: "United States" [MeSH] OR United States of America OR United States OR America OR U.S. OR US OR USA OR U.S.A.
Results: 8,614,646

Line 5: Line 3 + Line 4
Results: 212

Line 6: "Attitude" [MeSH] OR Barrier OR Barriers OR Facilitator OR Facilitators OR Determinant OR Determinants OR Factor OR Factors OR Influence OR Influences OR Attitude OR Attitudes
Results: 16,036,588

Line 7: Line 5 + Line 6
Results: 154

Filter for English
Results: 145

Filter for English+ 1990-2002
Results: 137

Date: 5/24/2002

Database: CINAHL

	**Concept: Vaccine Uptake**	**Concept: Refugee**	**Concept: U.S**	**Concept: Facilitators/Barriers**
**Thesaurus Terms/ Subheadings**	MH "Vaccination Coverage" OR<br>MH "Immunization" OR	MH "Refugee" OR	MH "United States" OR	MH "Attitude" OR
**Textwords**	"Vaccination" OR<br>"Vaccinations" OR<br>"Vaccine acceptance" OR<br>"Vaccination coverage" OR<br>"Vaccination coverage immunization" OR<br>"Immunization willingness" OR<br>"Immunization, active" OR<br>"Active immunization" OR<br>"Active immunizations" OR<br>"Immunizations, active"	"Refugee" OR<br>"Refugees" OR<br>"Political asylum seeker" OR<br>"Asylum Seeker, Political" OR<br>"Asylum Seekers, Political" OR<br>"Seekers, Political Asylum" OR<br>"Political refugees" OR<br>"Political refugee" OR<br>"Refugee, political" OR<br>"Refugees, political" OR<br>"Asylum Seeker" OR<br>"Asylum Seekers" OR<br>"Seeker, asylum" OR<br>"Seekers, asylum" OR<br>"Displaced persons" OR<br>"Displaced person" OR<br>"Person, displaced" OR<br>"Persons, displaced" OR<br>"Internally displaced persons" OR<br>"Displaced person, internally" OR<br>"Displaced persons, internally"	"United States of America" OR<br>"United States OR<br>"America" OR<br>"U.S." OR<br>"US" OR<br>"USA" OR<br>"U.S.A."	"Barrier" OR<br>"Barriers" OR<br>"Facilitator" OR<br>"Facilitators" OR<br>"Determinant" OR<br>"Determinants" OR<br>"Factor" OR<br>"Factors" OR<br>"Influence" OR<br>"Influences" OR<br>"Attitude" OR<br>"Attitudes"

		OR<br>“Internally displaced person”<br>OR<br>“Transients and migrants*” OR<br>“Migrant” OR<br>“Migrants”		

Line 1: MH “Vaccination Coverage” OR MH “Immunization” OR “Vaccination” OR “Vaccinations” OR “Vaccine acceptance” OR “Vaccination coverage” OR “Vaccination coverage immunization” OR “Immunization willingness” OR “Immunization, active” OR “Active immunization” OR “Active immunizations” OR “Immunizations, active“
Results: 49,606

Line 2: MH “Refugee” OR “Refugee” OR “Refugees” OR “Political asylum seeker” OR “Asylum Seeker, Political” OR “Asylum Seekers, Political” OR “Seekers, Political Asylum” OR “Political refugees” OR “Political refugee” OR “Refugee, political” OR “Refugees, political” OR “Asylum Seeker” OR “Asylum Seekers” OR “Seeker, asylum” OR “Seekers, asylum” OR “Displaced persons” OR “Displaced person” OR “Person, displaced” OR “Persons, displaced” OR “Internally displaced persons” OR “Displaced person, internally” OR “Displaced persons, internally” OR “Internally displaced person” OR “Transients and migrants*” OR “Migrant” OR “Migrants”
Results: 28,526

Line 3: Line 1 + Line 2
Results: 496

Line 4: MH “United States” OR “United States of America” OR “United States OR “America” OR “U.S.” OR “US” OR “USA” OR “U.S.A.”
Results: 392,274

Line 5: Line 3 + Line 4
Results: 70

Line 6: MH “Attitude” OR “Barrier” OR “Barriers” OR “Facilitator” OR “Facilitators” OR “Determinant” OR “Determinants” OR “Factor” OR “Factors” OR “Influence” OR “Influences” OR “Attitude” OR “Attitudes”
Results: 2,163,004

Line 7: Line 5 + Line 6
Results: 31

Filter for English
Results: 31

Filter for English + 1990-2002
Results: 30
Date: 5/25/2022

Database: Cochrane Library

	**Concept: Vaccine Uptake**	**Concept: Refugee**	**Concept: U.S.**	**Concept: Facilitators/Barriers**
**Thesaurus Terms/Subheadings**	“Vaccines” [MeSH] OR<br>“Immunization” [MeSH] OR	“Refugee” [MeSH] OR	“United States” [MeSH] OR	“Attitude” [MeSH] OR
**Textwords**	“Vaccination” OR<br>“Vaccinations” OR<br>“Vaccine acceptance” OR<br>“Vaccination coverage” OR<br>“Vaccination coverage immunization” OR<br>“Immunization willingness” OR<br>“Immunization, active” OR<br>“Active immunization” OR<br>“Active immunizations” OR<br>“Immunizations, active”	“Refugee” OR<br>“Refugees” OR<br>“Political asylum seeker” OR<br>“Asylum Seeker, Political” OR<br>“Asylum Seekers, Political” OR<br>“Seekers, Political Asylum” OR<br>“Political refugees” OR<br>“Political refugee” OR<br>“Refugee, political” OR<br>“Refugees, political” OR<br>“Asylum Seeker” OR<br>“Asylum Seekers” OR<br>“Seeker, asylum” OR<br>“Seekers, asylum” OR<br>“Displaced persons” OR<br>“Displaced person” OR<br>“Person, displaced” OR<br>“Persons, displaced” OR<br>“Internally displaced persons” OR<br>“Displaced person, internally” OR<br>“Displaced persons, internally” OR<br>“Internally displaced person” OR<br>“Transients and migrants*”	“United States of America” OR<br>“United States OR<br>“America” OR<br>“U.S.” OR<br>“US” OR<br>“USA” OR<br>“U.S.A.”	“Barrier” OR<br>“Barriers” OR<br>“Facilitator” OR<br>“Facilitators” OR<br>“Determinant” OR<br>“Determinants” OR<br>“Factor” OR<br>“Factors” OR<br>“Influence” OR<br>“Influences” OR<br>“Attitude” OR<br>“Attitudes”

		OR<br>"Migrant" OR<br>"Migrants"		

Line 1: "Vaccines" [MeSH] OR "Immunization" [MeSH] OR "Vaccination" OR "Vaccinations" OR "Vaccine acceptance" OR "Vaccination coverage" OR "Vaccination coverage immunization" OR "Immunization willingness" OR "Immunization, active" OR "Active immunization" OR "Active immunizations" OR "Immunizations, active"
Results: 25,227

Line 2: "Refugee" [MeSH] OR "Refugee" OR "Refugees" OR "Political asylum seeker" OR "Asylum Seeker, Political" OR "Asylum Seekers, Political" OR "Seekers, Political Asylum" OR "Political refugees" OR "Political refugee" OR "Refugee, political" OR "Refugees, political" OR "Asylum Seeker" OR "Asylum Seekers" OR "Seeker, asylum" OR "Seekers, asylum" OR "Displaced persons" OR "Displaced person" OR "Person, displaced" OR "Persons, displaced" OR "Internally displaced persons" OR "Displaced person, internally" OR "Displaced persons, internally" OR "Internally displaced person" OR "Transients and migrants*" OR "Migrant" OR "Migrants"
Results: 1,112

Line 3: Line 1 + Line 2
Results: 44

Line 4: "U.S." [MeSH] OR "United States of America" OR "United States OR "America" OR "U.S." OR "US" OR "USA" OR "U.S.A."
Results: 1,877,303

Line 5: Line 3 + Line 4
Results: 44

Line 6: "Attitude" [MeSH] OR "Barrier" OR "Barriers" OR "Facilitator" OR "Facilitators" OR "Determinant" OR "Determinants" OR "Factor" OR "Factors" OR "Influence" OR "Influences" OR "Attitude" OR "Attitudes"
Results: 397,786

Line 7: Line 5 + Line 6
Results: 30

Filter for English
Results: Cannot Filter for English in COCHRANE

Filter for English + 1990-2022
Results: 22
Date: 5/25/22

TOTAL RESULTS FROM ALL DATABASES: 189

TOTAL RESULTS AFTER DEDUPLICATION: 186

ROBIS: Tool to assess risk of bias in systematic reviews

Phase 1: Assessing relevance (Optional) The authors did not use Phase 1.

ROBIS is designed to assess the risk of bias in reviews with questions relating to interventions, aetiology, diagnosis and prognosis. State your overview/guideline question (target question) and the question being addressed in the review being assessed:

Intervention reviews:

Category	Target question (e.g. overview or guideline)	Review being assessed
Patients/Population(s):		
Intervention(s):		
Comparator(s):		
Outcome(s):		

For aetiology reviews:

Category	Target question (e.g. overview or guideline)	Review being assessed
Patients/Population(s):		
Exposure(s) and comparator(s):		
Outcome(s):		

For DTA reviews:

Category	Target question (e.g. overview or guideline)	Review being assessed
Patients):		
Index test(s):		
Reference standard:		
Target condition:		

For prognostic reviews:

Category	Target question (e.g. overview or guideline)	Review being assessed
Patients:		
Outcome to be predicted:		
Intended use of model:		
Intended moment in time:		

Does the question addressed by the review match the target question?	YES/NO/UNCLEAR

Phase 2: Identifying concerns with the review process

DOMAIN 1: STUDY ELIGIBILITY CRITERIA

Describe the study eligibility criteria, any restrictions on eligibility and whether there was evidence that objectives and eligibility criteria were pre-specified:

1.1 Did the review adhere to pre-defined objectives and eligibility criteria?	Y/PY/PN/N/NI
1.2 Were the eligibility criteria appropriate for the review question?	Y/PY/PN/N/NI
1.3 Were eligibility criteria unambiguous?	Y/PY/PN/N/NI
1.4 Were all restrictions in eligibility criteria based on study characteristics appropriate (e.g. date, sample size, study quality, outcomes measured)?	Y/PY/PN/N/NI
1.5 Were any restrictions in eligibility criteria based on sources of information appropriate (e.g. publication status or format, language, availability of data)?	Y/PY/PN/N/NI

Concerns regarding specification of study eligibility criteria LOW/HIGH/UNCLEAR

Rationale for concern:

DOMAIN 2: IDENTIFICATION AND SELECTION OF STUDIES

Describe methods of study identification and selection (e.g. number of reviewers involved):

2.1 Did the search include an appropriate range of databases/electronic sources for published and unpublished reports?	Y/PY/PN/N/NI
2.2 Were methods additional to database searching used to identify relevant reports?	Y/PY/PN/N/NI
2.3 Were the terms and structure of the search strategy likely to retrieve as many eligible studies as possible?	Y/PY/PN/N/NI
2.4 Were restrictions based on date, publication format, or language appropriate?	Y/PY/PN/N/NI
2.5 Were efforts made to minimise error in selection of studies?	Y/PY/PN/N/NI

Concerns regarding methods used to identify and/or select studies LOW/HIGH/UNCLEAR

Rationale for concern:

DOMAIN 3: DATA COLLECTION AND STUDY APPRAISAL

Describe methods of data collection, what data were extracted from studies or collected through other means, how risk of bias was assessed (e.g. number of reviewers involved) and the tool used to assess risk of bias:

3.1 Were efforts made to minimise error in data collection?	Y/PY/PN/N/NI
3.2 Were sufficient study characteristics available for both review authors and readers to be able to interpret the results?	Y/PY/PN/N/NI
3.3 Were all relevant study results collected for use in the synthesis?	Y/PY/PN/N/NI
3.4 Was risk of bias (or methodological quality) formally assessed using appropriate criteria?	Y/PY/PN/N/NI
3.5 Were efforts made to minimise error in risk of bias assessment?	Y/PY/PN/N/NI

Concerns regarding methods used to collect data and appraise studies LOW/HIGH/UNCLEAR

Rationale for concern:

DOMAIN 4: SYNTHESIS AND FINDINGS	
Describe synthesis methods:	
4.1 Did the synthesis include all studies that it should?	Y/PY/PN/N/NI
4.2 Were all pre-defined analyses reported or departures explained?	Y/PY/PN/N/NI
4.3 Was the synthesis appropriate given the nature and similarity in the research questions, study designs and outcomes across included studies?	Y/PY/PN/N/NI
4.4 Was between-study variation (heterogeneity) minimal or addressed in the synthesis?	Y/PY/PN/N/NI
4.5 Were the findings robust, e.g. as demonstrated through funnel plot or sensitivity analyses?	Y/PY/PN/N/NI
4.6 Were biases in primary studies minimal or addressed in the synthesis?	Y/PY/PN/N/NI
Concerns regarding the synthesis and findings<br>Rationale for concern:	LOW/HIGH/UNCLEAR

Y=YES, PY=PROBABLY YES, PN=PROBABLY NO, N=NO, NI=NO INFORMATION

Phase 3: Judging risk of bias

Summarize the concerns identified during the Phase 2 assessment:

Domain	Concern	Rationale for concern
1. Concerns regarding specification of study eligibility criteria	Low	The term "non-US born" was not included in the text words in the search strategy.
2. Concerns regarding methods used to identify and/or select studies	Low	The search was limited to 3 databases because the author's academic institution had a subscription to those three databases.
3. Concerns regarding methods used to collect data and appraise studies	Low	Not applicable.
4. Concerns regarding the synthesis and findings	Low	The heterogeneity was not addressed to the full extent. Additionally, the nature of the study did not allow the use of sensitivity analyses.

RISK OF BIAS IN THE REVIEW	
Describe whether conclusions were supported by the evidence:	
A. Did the interpretation of findings address all of the concerns identified in Domains 1 to 4?	Y/PY/PN/N/NI
B. Was the relevance of identified studies to the review's research question appropriately considered?	Y/PY/PN/N/NI
C. Did the reviewers avoid emphasizing results on the basis of their statistical significance?	Y/PY/PN/N/NI
Risk of bias in the review<br>Rationale for risk:	RISK: LOW/HIGH/UNCLEAR

Y=YES, PY=PROBABLY YES, PN=PROBABLY NO, N=NO, NI=NO INFORMATION
